# The Independent Domestication of Timopheev’s Wheat: Insights from Haplotype Analysis of the *Brittle rachis 1* (*BTR1-A*) Gene

**DOI:** 10.3390/genes12030338

**Published:** 2021-02-25

**Authors:** Moran Nave, Mihriban Taş, John Raupp, Vijay K. Tiwari, Hakan Ozkan, Jesse Poland, Iago Hale, Takao Komatsuda, Assaf Distelfeld

**Affiliations:** 1 Department of Evolutionary and Environmental Biology, Faculty of Natural Sciences and the Institute of Evolution, University of Haifa, Haifa 3498838, Israel; moranaway1@gmail.com; 2 School of Plant Sciences and Food Security, Tel Aviv University, Tel Aviv 6997801, Israel; 3 Department of Field Crops, Faculty of Agriculture, Cukurova University, Adana 01250, Turkey; mihribanesacer@gmail.com (M.T.); hozkan@cu.edu.tr (H.O.); 4 Wheat Genetics Resource Center, Department of Plant Pathology, Kansas State University, Manhattan, KS 66506, USA; jraupp@ksu.edu (J.R.); jpoland@ksu.edu (J.P.); 5 Department of Plant Science and Landscape Architecture, University of Maryland, College Park, MD 20742, USA; vijtiwari@gmail.com; 6 Department of Agriculture, Nutrition, and Food Systems, University of New Hampshire, Durham, NH 03824, USA; iago.hale@unh.edu; 7 National Institute of Agrobiological Sciences, Tsukuba, Ibaraki 305-8518, Japan; takao@affrc.go.jp; 8 Institute of Crop Science, National Agriculture and Food Research Organization (NARO), Tsukuba 305-8518, Japan

**Keywords:** brittle rachis, domestication, haplotype analysis, progenitor, wild emmer wheat, Timopheev’s wheat

## Abstract

*Triticum turgidum* and *T. timopheevii* are two tetraploid wheat species sharing *T. urartu* as a common ancestor, and domesticated accessions from both of these allopolyploids exhibit nonbrittle rachis (i.e., nonshattering spikes). We previously described the loss-of-function mutations in the *Brittle Rachis 1* genes *BTR1-A* and *BTR1-B* in the A and B subgenomes, respectively, that are responsible for this most visible domestication trait in *T. turgidum*. Resequencing of a large panel of wild and domesticated *T. turgidum* accessions subsequently led to the identification of the two progenitor haplotypes of the *btr1-A* and *btr1-B* domesticated alleles. Here, we extended the haplotype analysis to other *T. turgidum* subspecies and to the *BTR1* homologues in the related *T. timopheevii* species. Our results showed that all the domesticated wheat subspecies within *T. turgidum* share common *BTR1-A* and *BTR1-B* haplotypes, confirming their common origin. In *T. timopheevii*, however, we identified a novel loss-of-function *btr1-A* allele underlying a partially brittle spike phenotype. This novel recessive allele appeared fixed within the pool of domesticated Timopheev’s wheat but was also carried by one wild *timopheevii* accession exhibiting partial brittleness. The promoter region for *BTR1-B* could not be amplified in any *T. timopheevii* accessions with any *T. turgidum* primer combination, exemplifying the gene-level distance between the two species. Altogether, our results support the concept of independent domestication processes for the two polyploid, wheat-related species.

## 1. Introduction

Originating via hybridization among different progenitor species, allopolyploid wheat species are widespread due to their enhanced capacity for genetic adaptation relative to their diploid ancestors [1]. *Triticum urartu* [AA genome] underwent at least two independent spontaneous hybridizations with likely extinct forms of *Aegilops speltoides Tausch* (SS genome), one associated with the formation of *T. turgidum* ((2n = 4× = 28), BBAA genome) [2,3], and the other with the formation of *T. timopheevii* ((2n = 4× = 28), GGA^t^A^t^ genome) [4,5,6], also known as Timopheev’s wheat.

As the common ancestor of all economically important domesticated wheat species, *T. turgidum* drew much scientific attention; the genetic and morphological characteristics of this polyploid lineage were intensely investigated in an effort to understand the process of wheat evolution and domestication. A breakthrough in this regard was the scientific discovery of “wild emmer wheat” (WEW), *T. turgidum* L. subsp. *dicoccoides* (Korn. Ex Asch. & Graebn. Thell.) by Aaronsohn more than a century ago [7]. The sustained cultivation of *T. turgidum* in the Fertile Crescent is linked to the subsequent emergence of “domesticated emmer wheat” (DEW), *T. turgidum* L. subsp. *dicoccum* (Schrank ex Schübl.) Thell., the domesticated form of wild emmer wheat. Possessing nonbrittle spikes but tough glumes, domesticated emmer served as an important evolutionary step toward the development of the various fully domesticated (free-threshing) tetraploid wheat subspecies: Durum wheat (DW—*T. turgidum* L. subsp. *durum* (Desf.) Husn.), rivet wheat (TRG—*T. turgidum* L. subsp. *turgidum* (Desf.) Husn.), Khorasan wheat (TRN—*T. turanicum*), Polish wheat (POL—*T. polonicum*), Persian wheat (CRT*—T. carthlicum*), and Georgian emmer (PLC*—T. paleocolchicum*). Whether they evolved from a single, common, domesticated emmer ancestor or emerged independently from separate wild or domesticated emmer accessions is still unknown [8]. However, as members of the *T. turgidum* lineage, they all share the same BBAA genomic composition.

In contrast to *T. turgidum*, *T. timopheevii* received far less scientific attention. Thought to be domesticated later than emmer [9], *T. timopheevii* (Zhuk.) Zhuk. subsp. *timopheevii* is the domesticated form (“domesticated Timopheev’s wheat”—DTW or domesticated *timopheevii*) of the wild *T. timopheevii* (Zhuk.) Zhuk. subsp*. araraticum* Jakubz (“wild Timopheev’s wheat—WTW or *T. araraticum*), both possessing an GGA^t^A^t^ genomic composition. Geographically, *T. araraticum* grows from Armenia to Azerbaijan but also overlaps with wild emmer populations in southeastern Turkey, northern Iraq, and western Iran. In contrast, domesticated *timopheevii* is an endemic crop exclusively of western Georgia, to the north [9,10]. Using chloroplast DNA fingerprinting, the closest genetic similarity of *T. araraticum* accessions to domesticated *timopheevii* types was found among wild accessions collected from southern Turkey and northern Syria, implying *T. timopheevii* domestication might have taken place in these areas [9]. Archaeobotanical findings of early agrarian settlements discovered a different type of cultivated wheat other than domesticated emmer called “new glume wheat” (NGW), suggested to be related to *T. timopheevii* [11]. A recent study traced sequences from the G genome in NGW samples that were present across western Asia and Europe in the Neolithic and Bronze ages, indicating a wider spread than first thought and suggesting that domesticated *timopheevii* is of major importance to prehistoric Eurasian agriculture and not a just a minor crop restricted to western Georgia [12]. Supporting this, it was recently claimed that domestication of NGW took place in Transcaucasia and Anatolia in separate pathways [13], indicating the importance and widespread of the *timopheevii* species.

Although crosses between *T. turgidum* and *T. timopheevii* yielded F1 progeny, stable hybrid lines were not obtained, presumably due to failures in chromosome pairing, leading to infertility [4,14]. However, evidence of relatively good chromosome pairing in the F1 hybrids in some *T. timopheevii* × *T. turgidum* combinations were also reported [15]. Genome-wide variation between the two species was first demonstrated on the basis of large “species founder translocations” involving chromosomes 4A, 5A, 6A, 7B, 1G, and 4G [16,17]. Despite these significant distinctions between their genomes, *T. timopheevii* and *T. turgidum* share high homology with each other [18]. Indeed, cytogenetic examination suggests that genes can be transferred successfully between *T. turgidum* and *T. timopheevii* via direct crosses, though likely with a low success rate [19]. A more recent study, based on GBS data, supported the conclusion that *T. turgidum* and *T. timopheevii* are indeed distinct species [8], confirming the conclusions of earlier studies that the *T. turgidum* species evolved earlier [20].

Despite much progress in understanding the origin and evolution of *T. timopheevii*, the specific processes that shaped its domestication are still unknown. The hallmark trait of wheat domestication and arguably the most essential morphological change associated with this process is the transition from a brittle (shattering) to a nonbrittle (nonshattering) rachis [21]. During the domestication of *T. turgidum*, domesticated emmer acquired two recessive loss-of-function mutations in the A and B genome copies of the *Brittle Rachis 1* (*BTR1-A* and *BTR1-B*), resulting in a nonshattering (i.e., intact, harvestable) spike [22]. The causative mutation in *BTR1-A* is a 2 bp deletion in the coding sequence which causes a loss-of-function frame shift. In *BTR1-B*, loss of function is due to a 4 kbp insertion, 50 bp upstream of the stop codon.

In a previous study, we resequenced the *BTR1-A* and *BTR1-B* regions in a wide panel of wild and domesticated tetraploid wheat accessions and identified wild emmer individuals that represent likely progenitors (“founder stocks”) of domesticated emmer, with its stacked, nonfunctional *btr1-A* and *btr1-B* haplotypes [23]. Since mutations in the *BTR1* genes are associated with cereal domestication [21,22,24] we hypothesize that *T. timopheevii* was also domesticated through mutations in *BTR1* genes, but should carry distinct, novel *BTR1-A* and *BTR1-**G*** mutated alleles. To shed light on the presumably analogous process of *T. timopheevii* domestication, we present here an examination of the sequence variation associated with spike shattering (i.e., the brittle rachis trait) in various wild and domesticated *T. timopheevii* and *T. turgidum* accessions. Our results showed that domesticated *timopheevii* carries a novel *btr1-A* allele whose causative mutation is different from that underlying domesticated *T. turgidum* accessions. Using *T. turgidum* primers, the *BTR1-G* promoter region could not be amplified in any *T. timopheevii* accession, further illustrating the completely different origin of the two species and specifically the domesticated alleles in these different genomes.

## 2. Materials and Methods

### 2.1. Plant Material and Phenotyping

We evaluated a total of 57 accessions investigated in this study, 34 from the *T. timopheevi* species and 23 from the *T. turgidum* species (Table S1). Within *T. timopheevi*, 32 accessions are wild (*T. araraticum*) and 2 are domesticated (DTW). Within the *T. turgidum* accessions were representatives from six subspecies, including domesticated emmer (6)*, T. carthlicum* (5), *T. turanicum* (4), *T. turgidum* L. subsp. *turgidum* (4), *T. polonicum* (3), and *T. paleocolchicum* (1). In addition, the well-characterized wild emmer accession “Zavitan” [25] and durum wheat cv. “Svevo” were used as references. All accessions were grown in a greenhouse in four-liter pots at Tel Aviv University from January to May 2020. Based on five mature, senesced spikes from each accession, a visual assessment of brittle rachis phenotype was made according to three categories: (1) brittle—the spike completely shattered when harvested; (2) semi-brittle—only the upper half of the spike separated with a slight touch while the lower half remained intact; and (3) nonbrittle—the complete spike remained intact when harvested.

### 2.2. Amplification and Sequencing of the BTR1-A and BTR1-B Gene Regions

Using the methods described by [23], PCR amplification of the *BTR1-A* gene region was carried out using the forward primer 5′-TTGCTGTTGACAAAGGCCAG-3′ (located 93 bp upstream of the start codon) and the reverse primer 5′-TTTTCTCGTTCGCTACCACAC-3′ (located 912 bp downstream from the stop codon). Due to low sequencing quality at the 3′ end of the resulting 1596 bp amplicon, we developed and used an alternative reverse primer, 5′-TCGGGAGCTCATTTGACCTT-3′, located only 734 bp downstream from the stop codon. Amplification of the *BTR1-B* and *BTR1-G* gene regions was attempted with the same primers and PCR conditions were described by [23], encompassing a 2447 bp region starting 1856 bp upstream of the *BTR1-B* start codon and including 483 bp of the *BTR1-B* coding sequence. Despite designing 25 different primers specific to the promoter and the coding sequence of *BTR1-B* (Table S2), only the *T. turgidum* accessions (*BTR1-B*) were successfully amplified under these conditions.

### 2.3. BTR1-A/btr1-A Marker Development

To discriminate between wild and domesticated *T. timopheevii* alleles of *BTR1-A*, we developed a cleaved amplified polymorphic sequence (CAPS) marker using the forward primer 5′-GTCCGGTTCATGCTTCACAG-3′ and the reverse primer 5′-TGCCAATGTACGTTGCAAGT-3′. The 456 bp amplicon was digested with *AciI* restriction enzyme (New England Biolabs, Ipswich, MA, USA) and the resulting fragments were imaged via electrophoresis (2% agarose gel) to distinguish between the wild (456 bp) and domesticated (148 + 307 bp) *T. timopheevii* alleles.

### 2.4. Sequencing, Phylogenetic, Haplotypic, and Functional Analyses

Sequencing of the target regions was obtained using ABI 3500xl Genetic analyzer (Applied Biosystems, Foster City, CA, USA). This method of sequencing utilizes the BigDye Terminator Cycle Sequencing Kit (Thermo Fisher Scientific, Waltham, MA, USA) with fluorescent dye attached to each of the dideoxy terminators.

Amplified sequences of the two targeted regions were imported into Sequencher v5.4 (www.genecodes.com) and low-quality reads were manually removed. Sequence alignments were generated using ClustalW within MEGA 6 [26] and haplotypes were defined using DnaSP 5.10.01 [27]. Orthologous sequences from the *T. urartu* reference genome [28] were used as outgroups. Phylogenetic trees were constructed based on the neighbor-joining method using MEGA 6 [26], assuming uniform rates among sites. A bootstrap analysis (1000 replicates) was performed to provide confidence estimates for branch nodes. Translation to peptides based on the coding sequence of each accession was done with EMBOSS Transeq © EMBL 2020 (https://www.ebi.ac.uk/Tools/st/emboss_transeq/ (accessed on 31 January 2021)).

## 3. Results

### 3.1. BTR1-A Haplotype Analysis

**Figure 1:**
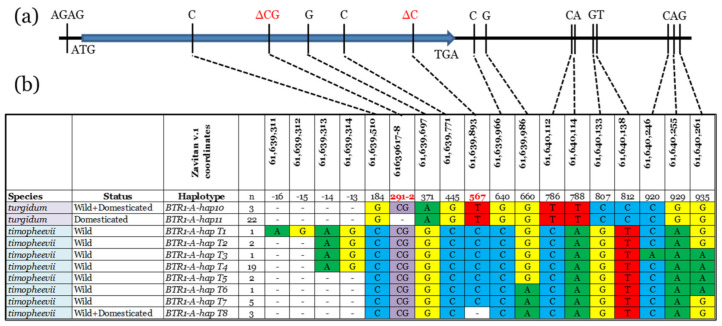
(**a**) Schematic showing the locations of the sequence polymorphisms used in the *BTR1-A* haplotype analysis. Highlighted in red font are two frameshift deletions in the *BTR1-A* coding sequence. (**b**) Among the materials included in this study, a total of 10 *BTR1-A* haplotypes were identified, two within the *T. turgidum* species and eight within the *T. timopheevii* species. In *T. timopheevii*, *BTR1-A-hapT7* is the closest wild haplotype to the domesticated haplotype *BTR1-A-hap T8*, differing only in the 1 bp frameshift deletion at position 567. The “Status” column refers to the historical domestication status according to the relevant seed source. Colors help to detect the differences.

High-quality sequence data of the *BTR1-A* region were obtained from 1244 bp amplicons, starting 27 bp upstream of the start codon and ending 627 bp downstream from the stop codon (chr. 3A 61,639,300–61,640,544, [22]), a region that overlaps with the one described by [23]. In addition to the previously described 2 bp frameshift deletion within the coding sequence (position 291–292), which separates wild from domesticated wheat [22], our analysis identified three other variants within the *BTR1-A* coding region, another 1 bp frameshift deletion (position 567), as well as nine SNPs located downstream from the coding region (Figure 1a).

This set of polymorphisms divided the studied accessions into ten haplotypic groups, two belonging to *T. turgidum* accessions and eight representing *T. timopheevii* accessions (Figure 1b). The Zavitan and Svevo sequences were identical to their corresponding reference genomes [22,29], and we used the same haplotype terminology as in Nave et al. [23], namely, *BTR1-A-hap10* and *BTR1-A-hap11,* to describe the Zavitan (wild emmer) and Svevo (durum wheat) haplotypes, respectively. Nearly all of the domesticated *T. turgidum* accessions carried the *BTR1-A-hap11* haplotype, except for DEW–10489 and DEW–10516, two accessions found to carry the wild-type *BTR1-A-hap10* haplotype (in combination with the loss-of-function *btr1-B* allele). Accession no. DEW–10489 originated from Jordan and exhibits some wild emmer characteristics (semi-brittle spike, wild emmer spike shape, and spikelet hairiness), calling into question its original classification by the genebank as domesticated emmer. Originating from Yemen, accession DEW–10516 possesses the typical domesticated emmer morphology, including a nonbrittle spike.

**Figure 2:**
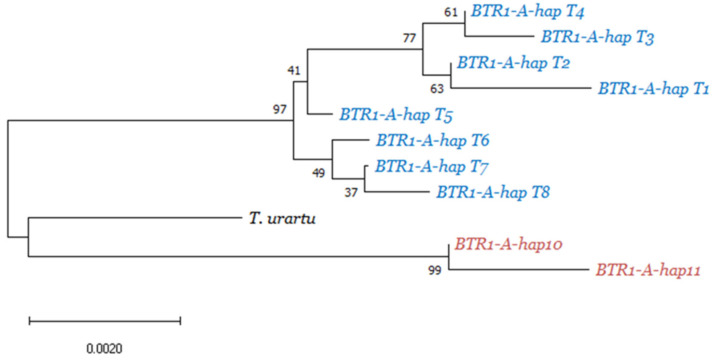
Phylogram based on *BTR1-A* variants showing clear differentiation between the *T. turgidum* (red) and *T. timopheevii* (blue) species, relative to the diploid *T. urartu* paraphyletic outgroup. The phylogram illustrates the close relationship between *T. araraticum* haplotype *BTR1-A-hapT7* and domesticated *timopheevii* haplotype *BTR1-A-hapT8*. Local bootstrap values after 1000 replicates are indicated at the nodes.

We identified eight distinct haplotypes among the *T. timopheevii* accessions (*BTR1-A-hapT1* to *T8*), with 31 of the 32 *T. araraticum* accessions belonging to the first seven (*BTR1-A-hapT1* to *T7*). Among the accessions included in this study, the largest *T. araraticum* haplotypic group is *BTR1-A-hapT4*, comprised of 19 accessions of broad geographic provenance, spanning Armenia, Azerbaijan, Iraq, and Iran (Table S1). Haplotypes *BTR1-A-hapT1, T2, T3, T5*, and *T6* are each represented by only one or two *T. araraticum* accessions, while *BTR1-A-hapT7* is carried by five *T. araraticum* accessions, all originating from Iraq. All 31 *T. araraticum* accessions from the first seven haplotypic groups exhibit a clear brittle rachis phenotype, as expected from wild plants. In contrast, the single *T. araraticum* from Iraq (WTW–102) carrying the *BTR1-A-hapT8* haplotype exhibits a semi-brittle rachis phenotype. Also in this haplotypic group are the two domesticated *timopheevii* accessions, with their nonbrittle rachis spikes. The *BTR1-A-hapT8* haplotype is distinguished by a cytosine deletion at position 567 of the coding sequence, 22 bp upstream of the stop codon. This single bp frameshift mutation is the only polymorphism that differentiates *BTR1-A-hapT8* from its most closely related haplotype *BTR1-A-hapT7* (Figure 1b), and we verified the deletion using a CAPS marker. A phylogenetic tree based on these results (Figure 2) showed a clear separation of the *T. turgidum* and *T. timopheevi* species, as well as two separate branches within each.

From analysis of the translated coding sequences of the *BTR1-A* haplotypes, we identified three amino acid polymorphisms (positions 62, 124 and 149) between the *T. timopheevii* accessions and the wild emmer Zavitan reference. For *BTR1-A-hapT8*, carrying the unique cysteine deletion, all seven amino acids (positions 190–197) downstream of the frameshift were different, compared with other haplotypes.

### 3.2. BTR1-B Haplotype Analysis

We used a similar approach to study the haplotypic variation of the *Btr1-B* and *Btr1-G* genes. In agreement with previous analyses, all domesticated *T. turgidum* accessions were found to carry *BTR1-B-hap8*, the domesticated haplotype described by Nave et al. [23]. Unlike the *BTR1-A* case, none of the *T. araraticum* or domesticated *timopheevii* accessions could be amplified for the *BTR1-G* promoter region using any primer combinations based on available *T. turgidum* references (Table S2). However, most of the coding sequence region was successfully amplified from *T. timopheevi* accessions using the forward primer 5′-CGCAATGGAAGAAGATGTACCA-3′ (located 14 bp upstream of the start codon in *T. turgidum*) and various reverse primers (Table S2). Since the *Btr1-B* region in *T. turgidum* is a complex of gene duplication clusters (with four *Btr1-B* copies) [22,29]; given the partial sequence data obtained for the *T. timopheevi* accessions, misidentifying the correct *Btr1-B* ortholog was possible. Therefore, in this study, the haplotype analysis was exclusively focused on the *BTR1-A* gene for the *T. timopheevi* accessions.

## 4. Discussion

Haplotype analyses of the *BTR1-A* gene region reconfirmed the clear separation between the *T. turgidum* and the *T. timopheevii* at the gene-level, in agreement with previous observations of the large genetic distance between them [5,8,9,20]. Consistent with this observation, the lack of consistent amplification for the *BTR1* locus from *T. timopheevii* G subgenome likewise supported the significant genetic distance between *T. turgidum* and *T. timopheevii*. New insight into the comparative histories of domestication within these lineages was afforded by the *BTR1-*A haplotype analysis, however. Specifically, the causative mutation in *BTR1-A* that underlies the domestication syndrome of emmer was discovered to be different from that carried by domesticated accessions of Timopheev’s wheat, a result which suggests independent domestication pathways for the two species.

### 4.1. The Triticum Turgidum Lineage

Previous haplotype analyses [22,23] suggested that the recessive, loss-of-function *btr1-A* and *btr1-B* alleles are fixed in all domesticated emmer accessions. Here, however, we discovered two domesticated emmer accessions (DEW–10489 and DEW–10516) carrying the wild-type *Btr1-A* allele (*BTR1-A-hap10*) in combination with the domesticated *btr1-B* allele. *BTR1-A-hap10* found in these two accessions and shared by some wild emmer accessions was identified to be the closest haplotype to *BTR1-A-hap11*, the haplotype carried by all other domesticated emmer lines studied to date. *BTR1-A-hap10* is the basal haplotype present in most wild emmer populations from both Northern and Southern Levant [23]; therefore, it is possible that accessions DEW–10489 and DEW–10516 represent intermediate accessions that acquired only the loss-of-function allele of the *BTR1-B* gene (*btr1-B*), but not the causative mutation of *BTR1-A* (*btr1-A*).

Such accessions might thus be viewed as “missing links” in emmer wheat domestication, exhibiting phenotypes that fall intermediately along the domestication spectrum depending on their overall, genome-wide loads of domesticated alleles. Understood in this way, the intermediate brittle-rachis phenotype of DEW–10489 and the apparently nonbrittle phenotype of DEW–10516 may represent different stages of nonbrittle rachis allele fixation during the process of domestication. One alternative explanation is that these accessions could instead be progeny from spontaneous crosses between wild and domesticated accessions. Under this scenario, we hypothesize that accession DEW–10489 may represent a progeny of such a cross that was not (spontaneously) backcrossed to domesticated wheat, or lost many domestication-related alleles, hence retaining some characteristics of wild emmer. In contrast, accession DEW–10516 may represent a hybrid progeny that somehow was backcrossed to domesticated emmer (once or more), hence the typical domesticated phenotype. Further study is needed to clarify why the effect of the active *Btr1-A* allele on rachis brittleness was not detected in DEW–10516.

Except for accessions DEW–10489 and DEW–10516, all tested *T. turgidum* accessions from the six domesticated subspecies (DEW, CRT, TRN, TRG, POL, and PLC) were found to carry the domesticated haplotypes in both the A and B subgenomes (*BTR1-A-hap11*/*BTR1-B-hap8*) that contain the loss-of-function alleles of the *BTR1-A* and *BTR1-B* genes. These results strengthen the conclusion that domesticated accessions within the *T. turgidum* lineage have a monophyletic origin [22,23].

### 4.2. Triticum Timopheevii Domestication

Haplotype analysis of wheat domestication gene *BTR1-A* revealed seven *T. araraticum* haplotypes (*BTR1-A-hapT1 to T7*) and one haplotype (*BTR1-A-hapT8*) carried by all domesticated accessions and one wild accession (WTW–102). The single bp frameshift mutation found exclusively in the coding sequence of the *BTR1-A-hapT8* haplotype is responsible for changing seven amino acids in the C-terminal end of the protein. Such a modification is expected to alter the function of the protein [30], potentially contributing to the nonbrittle rachis phenotype exhibited by the domesticated *timopheevii* accessions in this study. The existence of two independent, functional mutations in the *BTR1-A* genes within the *T. turgidum* and *T. timopheevii* species supports that the *turgidum* and *timopheevii* domestication processes were distinct from one another and occurred post polyploidization.

Observed in 19 out of 32 *T. araraticum* accessions, *BTR1-A-hapT4* is suggested by its abundance and wide geographic distribution to be the most ancestral (basal) *T. araraticum* haplotype. A majority of the *T. araraticum* accessions in this work were collected from Iraq (21 out of 32), and most of the haplotypes contained Iraqi representatives (six out of seven), the latter point suggesting that this region may be the center of diversity for *T. timopheevii*. Moreover, *BTR1-A-hapT7* (Figure 2), the closest wild haplotype to the domesticated *BTR1-A-hapT8* haplotype, consists only of *T. araraticum* accessions of Iraqi provenance. These results suggest that the *T. araraticum* founder stock carried the *BTR1-A-hapT7* haplotype and pinpoints Iraq as the likely place of *T. timopheevii* domestication, conflicting with previous results that point to northern Syria and southern Turkey as the domestication regions [9], but supporting relatively new evidence of a larger geographical distribution of domesticated *timopheevii* than previously thought [12,13].

We failed to amplify the *BTR1-G* promoter region from any *T. timopheevi* accession, suggesting that this gene is highly polymorphic between the *T. turgidum* and the *T. timopheevii* species. *Aegilops speltoides* is the G-genome donor for the formation of *T. timopheevii*, and this wild species carries an intact *Btr1* homolog [31], implying that *Btr1* may have mutated in *T. timopheevii* after polyploidization. Because a reference genome of at least one *T. timopheevii* accession is likely required to resolve this point, we focused here only on the *BTR1-A* gene.

In addition to the strongly determinant *BTR1-A* and *BTR1-B* genes, the nonbrittle rachis phenotype in *T. turgidum* lineage is associated with several other smaller effect loci (QTLs) as well [22], something that is also likely the case in the *T. timopheevii* lineage. Accordingly, the nonbrittle phenotypes of DTW–2729 and DTW–2804 may be explained by the combination of the domesticated *BTR1-A-hapT8* haplotype with as-yet unidentified mutation(s) in the A^t^ and/or G subgenomes of domesticated *timopheevii*. We suggest that the semi-brittle accession WTW–102 from Iraq, also carrying the *BTR1-A-hapT8* haplotype, may represent a missing link in *T. timopheevii* domestication, analogous to DEW–10489 and DEW–10516 within the *T. turgidum* lineage.

## 5. Conclusions

This is the first report of the likely nonfunctional *btr1-A* allele that contributes to the domesticated nonbrittle phenotype in *T. timopheevii*, noteworthy due to its difference from the domesticated *T. turgidum* loss-of-function *btr1-A* allele. While extended haplotype analysis confirmed the clear distinction between the *T. timopheevii* and *T. turgidum* lineages, it is the unique single bp frameshift in domesticated *timopheevii* that suggests an independent domestication process for Timopheev’s wheat. In seeking to understand the domestication processes for these two lineages, we presented the concept of “missing link” accessions that carry only one loss-of-function allele and exhibit intermediate brittle rachis phenotypes. Our results point to Iraq as an important center of divergence for *T. timopheevii* and the region where domestication probably occurred. We also found that all six domesticated *T. turgidum* subspecies carry the same *BTR1-A* and *BTR1-B* haplotypes, suggesting a shared domestication pathway.

## Data Availability

Data is contained within the article or supplementary material.

## Supplementary Materials

The following are available online at https://www.mdpi.com/2073-4425/12/3/338/s1: Table S1: Distribution and phenotypic assessment of the 57 genotypes used for haplotype analysis; Table S2: List of primers designed to amplify the promoter and the coding sequence of *BTR1-B*.
